# Unemployment associated with 50% higher risk of death in heart-failure patients

**Published:** 2017

## Introduction

Unemployment is associated with a 50% higher risk of death in patients with heart failure, according to research presented recently at Heart Failure 2017 and the 4th World Congress on Acute Heart Failure. The observational study in more than 20 000 heart-failure patients found that not being employed was linked with a greater likelihood of death than a history of diabetes or stroke.

‘The ability to hold a job brings valuable information on wellbeing and performance status’, said lead author Dr Rasmus Roerth, a physician at Copenhagen University Hospital, Denmark. ‘And workforce exclusion has been associated with increased risk of depression, mental health problems and even suicide.’

‘In younger patients with heart failure, employment status could be a potential predictor of morbidity and mortality’, he continued. ‘If that was the case, employment status could help to risk stratify young heart-failure patients and identify those needing more intensive rehabilitation.’

This study compared the risks of all-cause death and recurrent heart-failure hospitalisation in patients with heart failure, according to whether they were employed at baseline or not. Using the unique personal identification number assigned to all residents in Denmark, individual data were linked from nationwide registries on hospitalisation, prescribed medication, education level, public welfare payments and death.

The study included all patients of working age (18 to 60 years) with a first hospitalisation for heart failure in Denmark between 1997 and 2012. Of the 21 455 patients with a first hospitalisation for heart failure, 11 880 (55%) were part of the workforce at baseline.

During an average follow up of 1 005 days, 16% of employed and 31% of unemployed patients died, while 40% of employed and 42% of unemployed patients were rehospitalised for heart failure.

After adjusting for age, gender, education level and co-morbidities, heart-failure patients unemployed at baseline had a 50% increased risk of death and 12% increased risk of rehospitalisation for heart failure compared to those who were employed. Not being part of the workforce was associated with a higher likelihood of death than a history of diabetes or stroke.

Dr Roerth said: ‘We found that heart-failure patients out of the workforce at baseline had a higher risk of death. Not being part of the workforce was associated with a risk of death comparable to that of having diabetes or stroke. Those without a job also had an increased risk of recurrent heart-failure hospitalisation.’

Dr Roerth said the exact mechanism on how employment status may affect mortality is complex and most likely multifactorial. ‘The ability to work can be seen as a measure of performance status and be interpreted as whether patients meet the physical requirements of a full time job or not’, he said.

But he added: ‘Employment status is more than just a physical measurement as it also has an influence on quality of life, and has been shown to be important for mental health and wellbeing. Thus, both from a physical and psychological point of view it makes sense to include employment status in the evaluation of young heart-failure patients’ prognosis.’

Dr Roerth said it was perhaps not surprising that employment status has importance for prognosis. ‘But the observation that employment status is associated with an increased risk of death comparable to that of many other co-morbidities such as diabetes and stroke is notable’, he said.

In terms of implications of the findings, Dr Roerth said workforce exclusion could be used to identify heart failure patients at risk of poor outcomes and that efforts to get patients back into work might be beneficial.

He said: ‘It could be highly valuable to assess employment status and actually think of workforce exclusion as a prognostic marker in line with suffering from serious chronic diseases. Knowledge on why workforce exclusion has happened for the individual patient might lead to ideas on how it can be prevented – for example with more intensive rehabilitation, physical activity, psychological treatment, or a different job.’
